# Toll-like receptor 1 predicts favorable prognosis in pancreatic cancer

**DOI:** 10.1371/journal.pone.0219245

**Published:** 2019-07-17

**Authors:** Mira Lanki, Hanna Seppänen, Harri Mustonen, Jaana Hagström, Caj Haglund

**Affiliations:** 1 Department of Surgery, University of Helsinki and Helsinki University Hospital, Helsinki, Finland; 2 Translational Cancer Medicine Research Program, University of Helsinki and Helsinki University Hospital, Helsinki, Finland; 3 Department of Pathology, University of Helsinki and Helsinki University Hospital, Helsinki, Finland; German Cancer Research Center, GERMANY

## Abstract

**Background:**

The link between inflammation and carcinogenesis is indisputable. In trying to understand key factors at play, cancer research has developed an interest in the toll-like receptors (TLRs), which have shown signs of having prognostic value in various adenocarcinomas. We began investigating the expression of toll-like receptors 1, 3, 5, 7, and 9 to evaluate their prognostic value of patients with pancreatic ductal adenocarcinoma (PDAC).

**Methods:**

We collected tumor biopsies from 154 stage I-III PDAC patients surgically treated at Helsinki University Hospital between 2002 and 2011, excluding patients undergoing neoadjuvant therapy. We used tissue microarray slides and immunohistochemistry to assess expression of TLRs 1, 3, 5, 7, and 9 in PDAC tissue. Immunopositivity scores and clinicopathological characteristics were subjected to Fisher’s exact test or the linear-by-linear association test. For the survival analysis, we applied the Kaplan-Meier method and log-rank test, and the Cox regression proportional hazard model served for univariate and multivariate analyses.

**Results:**

Strong TLR1 expression was observable in 60 (39%), strong TLR3 in 48 (31%), strong TLR5 in 58 (38%), strong TLR7 in 14 (9%), and strong TLR9 in 22 (14%) patients. The multivariate analysis showed strong TLR1 expression to associate with better survival than moderate, low, or negative expression (HR = 0.68; 95% CI 0.47–0.99; p = 0.044). Additionally, those few patients with tumors negative for TLR1, TLR3, TLR7, or TLR9 fared poorly (HR = 2.41; 95% CI 1.31–4.43; p = 0.005; n = 13).

**Conclusion:**

Strong TLR1 expression suggested better prognosis in PDAC patients, whereas negative expression of TLR1, TLR3, TLR7, or TLR9 was a sign of poor prognosis.

## Introduction

Pancreatic ductal adenocarcinoma (PDAC) is infamous for its aggressiveness. In western countries, it is the fourth leading cause of cancer-related death, with its numbers increasing [1,2]. The problem arises from mild initial symptoms leading to a late diagnosis, and this cancer’s tendency toward early metastasis, meaning that only 15 to 20% of patients undergo a resection with combined oncological treatment, the only chance for a cure [3–5]. Variation in survival time suggests a need for better prognostic markers, which may also lead to uncovering mechanisms affecting survival.

Chronic local inflammation is one of the best-known risks for cancer development, likely due to tumor-favoring microenvironmental changes [6]. The role of local inflammation is complicated, as some characteristics favor tumor progression, whereas others prevent it [7].

The toll-like receptors (TLRs) are key mediators of local inflammation. They recognize unfamiliar molecules and activate the NF-κB pathway, leading to a release of inflammatory cytokines and interferon-β; this enhances tissue regeneration and cell proliferation [8,9].

TLRs occur primarily in immunocytes, but the gut, respiratory, and ovarian epithelia also possess the potential to express them. Numerous carcinomas derived from these tissues express TLRs in abundance [10]. NF-κB is well-known for its crucial role in cancer development [11], but the role of the TLRs in carcinogenesis seems inadequately understood. Recently, PDAC progression has been associated with the presence of intestinal microbiota [12]. This is noteworthy, as it provides a physiological link between PDAC and TLR expression for the first time.

Prognostic implications of cancerous TLR expression vary. Most typically, TLR expression has been associated with worse prognosis, as in ovarian, lung, colorectal, and tongue carcinomas [13–16], but also association with better prognosis has been evident, as in esophageal carcinoma [17]. Our earlier study showed that strong TLR2 and TLR4 expression predict better prognosis for local PDAC [18]. In the current study, we expanded our focus to analyze the prognostic role of toll-like receptors 1, 3, 5, 7, and 9 in PDAC.

## Materials and methods

The data originated from consecutive PDAC patients who underwent surgery in 2002–2011 in the Department of Surgery, Helsinki University Hospital, Finland. We excluded 21 patients receiving neoadjuvant therapy, 7 patients because of re-diagnosis of stage-IV disease, and 4 patients with insufficient available data, leaving 154 patients for analysis. Our sources for the survival data and cause of death were hospital records, the Finnish Population Registry, and Statistics Finland.

Surgical tumor samples remained in storage, fixed in formalin and embedded in paraffin in the Department of Pathology, Helsinki University Hospital. Expert pathologists reconfirmed the histopathological diagnosis of PDAC. For the TLR evaluation process, we prepared multipunch tissue microarray blocks (TMAs) from the tumor samples, selecting representative regions on hematoxylin-eosin-stained slides, and then puncturing 1.0-mm cores from the tumor sample at the appropriate location. We gathered six spots for every patient to ensure that each TMA block was most representative of the tumors’ properties. For the TMA technique we utilized a semiautomatic tissue microarrayer (Tissue Arrayer 1, Beecher Instruments Inc., Silver Spring, MD, USA).

We then cut the TMA blocks into 4-μm sections and deparaffinized and rehydrated them. We treated the slides in an appropriate buffer for 20 minutes at 98°C and then incubated them at room temperature for 18 hours in the primary antibody solution. For TLR1, Tris-HCL (pH 8.5) served as the buffer, whereas for TLRs 3, 5, 7, and 9, the buffer was Tris-EDTA (pH 9.0). For TLR1, TLR3, and TLR7, we used rabbit polyclonal antibodies: TLR1 (H-90): sc-30000 (1:100) (Santa Cruz Biotechnology, Santa Cruz, CA, USA), TLR3 (H-125): sc-10740 (1:50) (Santa Cruz Biotechnology), and TLR7 NBP2-24906 (1:500) (Novus Biologicals, Littleton, CO, USA). For TLR5 and TLR9, we used mouse monoclonal antibodies: TLR5 NBP2-24787 (1:100) (Novus Biologicals) and TLR9 (26C593): sc-52966 (1:300) (Santa Cruz Biotechnology).

We evaluated the expression of the TLRs 1, 3, 5, 7, and 9 in the PDAC tissue by their staining intensity. Two independent observers (M.L. and J.H.) evaluated the staining score, and in cases with differing values, consensus was achieved through discussion. The staining intensity rate was on a scale from 0 to 3, where 0, 1, 2, and 3 indicated negative, mild, moderate, and strong staining. Only staining of epithelial tumor cells influenced the score. If a patient received differing scores for different samples, the choice was the maximal score for analysis. For TLR5, we assessed cytoplasmic and nuclear staining separately. For each individual TLR analysis, TLR scores 0 and 1 were combined for statistical purposes. Samples of skin and pharyngeal and palatine tonsils, known to be positive for the TLRs, served as positive controls for each staining series.

We applied Fisher’s exact test and the linear-by-linear association test to compare staining scores with clinicopathological parameters. The Kaplan-Meier method and the log-rank test served for survival analysis. For univariate and multivariate analyses, we utilized the Cox regression proportional hazard model. (The Cox model assumption of constant hazard ratio over time was tested by including time-dependent covariates separately for each variable.)

This study has the approval of the Helsinki University Hospital Surgical Ethics Committee (document number HUS 226/E6/06, additional petition TMK02 § 66/2013). The Finnish National Supervisory Authority for Welfare and Health gave permission to use archive material for this retrospective study without individual informed consent from the patients (document number 1004/06.01.03.01/2012). The staining intensity was evaluated without access to patient data. Statistical analysis was performed on anonymous but complete patient data including clinic-pathological and survival data.

## Results

### Immunohistochemistry

We successfully scored TLR1 for 154 patients, and TLR3, TLR5, TLR7, and TLR9 for 153 patients, with all missing TLR scores coming from the same patient.

**Fig 1:**
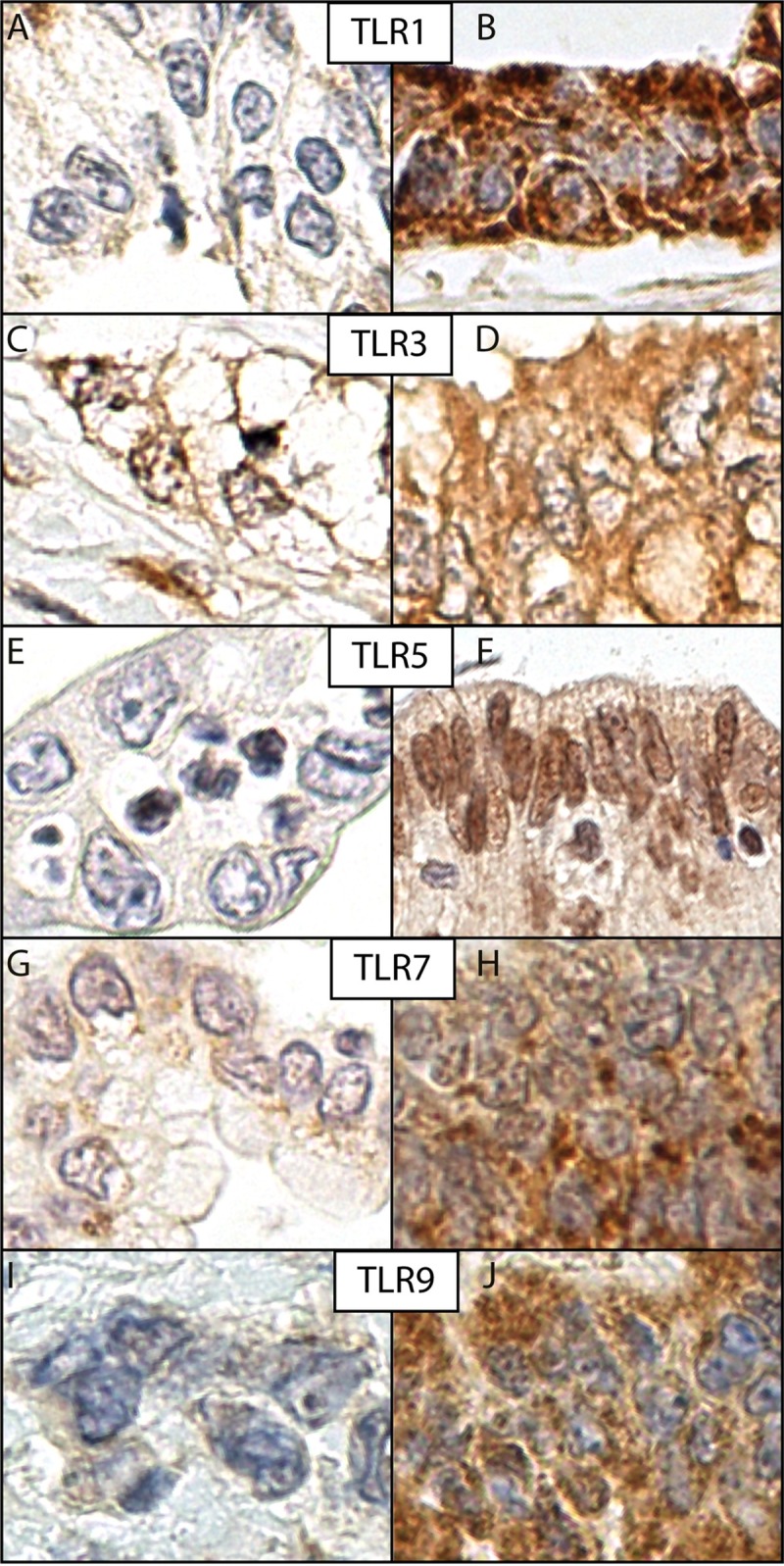
Staining patterns. From top to bottom: Staining in toll-like receptors 1, 3, 5, 7, and 9. Negative staining in A, C, E, G, and I; positive staining in B, D, F, H, and J.

For TLRs 1, 3, 7, and 9, immunopositivity was detectable in the cytoplasm with no notable membranous or nuclear positivity. TLRs 1, 7, and 9 exhibited some granular staining in the cytoplasm, whereas TLR3 stained more evenly. TLR5 expression differed from that of the rest of the TLRs by its distinctive nuclear positivity, and by detectable positivity in the cytoplasm in some samples. Occasionally, even the healthy epithelium stained positively. (Fig 1)

We observed strong TLR1 expression in 60 (39%) patients, strong TLR3 in 48 (31%), strong TLR5 in 58 (38%), strong TLR7 in 14 (9.1%), and strong TLR9 in 22 (14%). Negative immunoexpression was particularly rare for all receptors except TLR5. Negative TLR1 was observable in 3 (1.9%) patients, negative TLR3 in 2 (1.3%), negative TLR5 in 30 (19%), negative TLR7 in 5 (3.2%), and negative TLR9 in 7 (4.5%). Many of these negative TLR scores coincided in the same patients: 13 tumors showed negativity in one or more of the receptors TLR1, TLR3, TLR7, or TLR9. (Table 1)

**Table 1:** Toll-like receptor (TLR) expression distribution (%).

	Strong	Moderate	Low	Negative
**TLR1**	60 (39)	74 (48)	17 (11)	3 (2)
**TLR3**	48 (31)	79 (52)	24 (16)	2 (1)
**TLR5,** **cytoplasmic**	2 (1)	49 (32)	74 (48)	28 (18)
**TLR5,** **nuclear**	58 (38)	39 (25)	26 (17)	30 (20)
**TLR7**	14 (9)	75 (49)	59 (39)	5 (3)
**TLR9**	22 (14)	78 (51)	46 (30)	7 (5)

Strong and moderate TLR1 expression was more common among older patients (≥ 65 years; p = 0.016). TLR1 intensity associated with no other clinicopathological parameters: gender, cancer stage, lymph-node ratio, tumor size, or microscopic invasion. Negativity for TLRs 1, 3, 7, or 9 associated with no clinicopathological parameter. (Tables 2 and 3)

**Table 2:** Association between toll-like receptor 1 (TLR1) immunoexpression and clinicopathological parameters (%).

TLR1 expression	Strong	Moderate	Low or negative	P value
**N (%)**	**60 (39)**	**74 (48)**	**20 (13)**	
**Age, years**				
< 65	13 (22)	19 (26)	11 (55)	**0.016**
≥ 65	47 (78)	55 (74)	9 (45)	
**Gender**				
Male	28 (47)	48 (65)	9 (45)	0.474
Female	32 (53)	26 (35)	11 (55)	
Stage*				
I	12 (20)	8 (11)	5 (25)	0.402
II	36 (60)	45 (61)	9 (45)	
III	12 (20)	21 (28)	6 (30)	
**Lymph-node ratio**				
< 20%	47 (78)	57 (77)	14 (70)	0.476
≥ 20%	12 (20)	17 (23)	6 (30)	
Missing data	1 (2)	0 (0)	0 (0)	
**Tumor size**				
≤ 30 mm	29 (48)	34 (46)	8 (40)	0.468
> 30 mm	28 (47)	38 (51)	12 (60)	
Missing data	3 (5)	2 (3)	0 (0)	
**Perivascular invasion**				
Yes	14 (23)	21 (28)	8 (440)	0.222
No	35 (58)	38 (51)	10 (50)	
Missing data	11 (18)	15 (20)	2 (10)	
**Perineural invasion**				
Yes	36 (60)	51 (69)	14 (70)	0.448
No	14 (23)	12 (16)	4 (20)	
Missing data	10 (17)	11 (15)	2 (10)	

Associations evaluated by Fisher’s exact test or linear-by-linear association test.

*Staging according to AJCC 8^th^ edition.

**Table 3:** Association between negativity in toll-like receptors 1, 3, 7, and 9 and clinicopathological characteristics (%).

TLR status	All positive, n = 140	Negative in at least one receptor, n = 13	P value
**N (%)**	**140 (92%)**	**13 (8%)**	
**Age, years**			
< 65	38 (27)	5 (38)	0.519
≥ 65	102 (73)	8 (62)	
**Gender**			
Male	75 (54)	9 (69)	0.385
Female	65 (46)	4 (31)	
Stage*			
I	24 (17)	1 (8)	0.495
II	82 (59)	8 (62)	
III	34 (24)	4 (31)	
**Lymph-node ratio**			
< 20%	108 (77)	10 (77)	1.000
≥ 20%	31 (22)	3 (23)	
Missing data	1 (1)	0 (0)	
**Tumor size**			
≤ 30 mm	65 (46)	6 (46)	1.000
> 30 mm	70 (50)	7 (54)	
Missing data	5 (4)	0 (0)	
**Perivascular invasion**			
Yes	37 (26)	6 (46)	0.185
No	77 (55)	5 (38)	
Missing data	26 (19)	2 (15)	
**Perineural invasion**			
Yes	93 (66)	8 (62)	0.708
No	26 (19)	3 (23)	
Missing data	21 (15)	2 (15)	

Associations evaluated by Fisher’s exact test or linear-by-linear association test.

*Staging according to AJCC 8^th^ edition.

**Fig 2:**
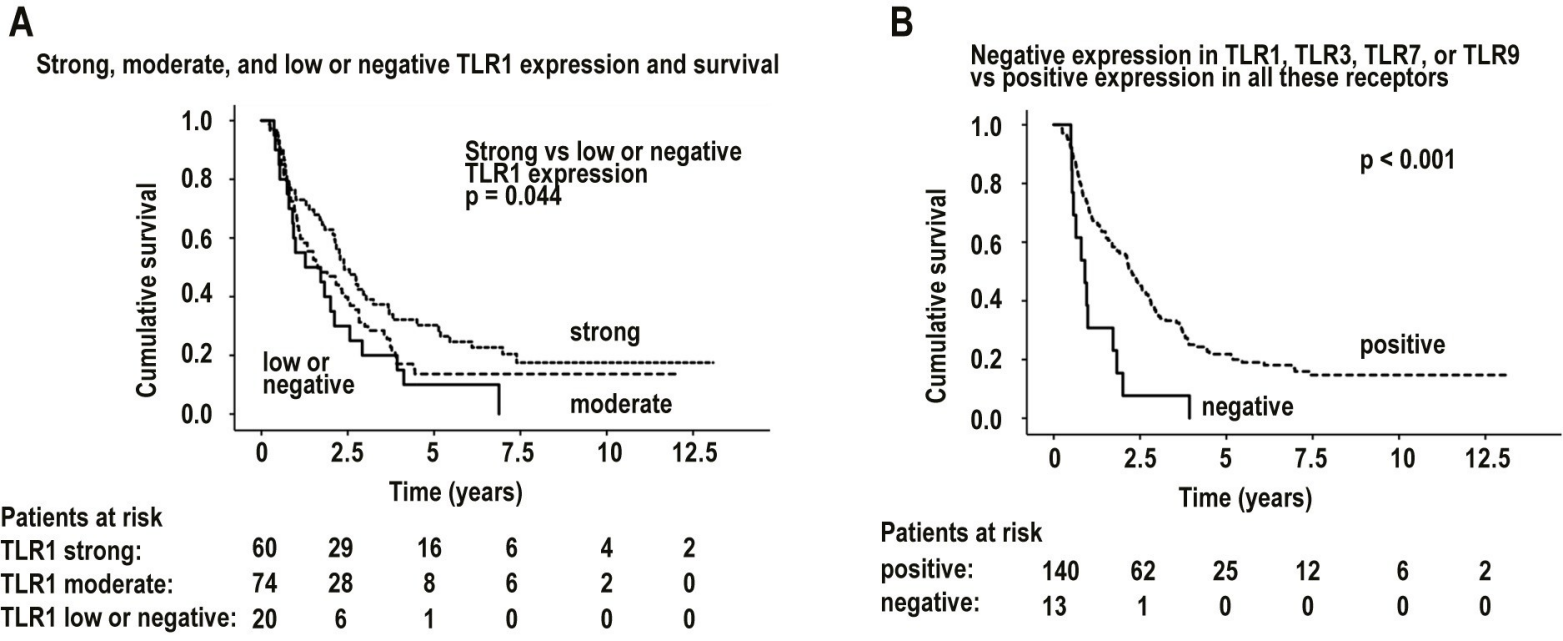
Kaplan-Meier curves. A: Survival in all patients, grouped by toll-like receptor 1 (TLR1) expression intensity. B: Survival in all patients, grouped by positive and negative expression of toll-like receptors 1, 3, 7, or 9 (TLR1, TLR3, TLR 7, TLR9).

According to univariate analysis, those patients with strong TLR1 tumor expression had better overall disease-specific survival than did patients whose TLR1 expression was low or negative (median survival time 2.40 (95% CI 1.78–3.01; n = 154) years and 1.27 (95% CI 0.00–2.87) years; log-rank with Sidak adjustment for multiple comparisons, p = 0.0439) (Fig 2).

Furthermore, multivariate analysis revealed strong TLR1 expression to associate with better overall disease-specific survival (strong vs moderate, low, or negative TLR1 expression; HR = 0.68; 95% CI 0.47–0.99; p = 0.044; n = 154; adjusted for age, gender, stage, lymph-node ratio, and adjuvant therapy). This finding was more pronounced among patients who received no adjuvant therapy (HR = 0.51; 95% CI 0.29–0.91; p = 0.023; n = 74). (Table 4). Those who did receive adjuvant therapy showed no sign of any survival benefit related to strong TLR1 expression (HR = 1.00; 95% CI 0.59–1.69; p = 0.989; n = 80).

**Table 4:** Multivariate sub-group analysis of pancreatic ductal adenocarcinoma patients without post-operative adjuvant therapy.

Multivariate analysis, patients without adjuvant therapy, n = 74				
		Hazard ratio	95% CI	P value
**Age, years**				
< 65		1.00		
≥ 65		1.47	0.73–2.96	0.276
**Gender**				
Male		1.00		
Female		1.14	0.67–1.95	0.630
Stage*	**Lymph-node ratio**			
IA–IIA		1.00		**0.004**
IIB, III	< 20%	1.77	0.97–3.26	0.065
IIB, III	≥ 20%	3.37	1.63–7.00	**0.001**
**TLR1**				
Negative, mild, or moderate		1		
Strong		0.51	0.29–0.91	**0.023**

Multivariate Cox analysis. Multivariate analysis adjusted for age, gender, stage, lymph-node ratio, and post-operative adjuvant therapy.

*Staging according to AJCC 8^th^ edition.

Another phenomenon in our study was the exceptionally poor survival of patients with negative scores for one or more of the receptors TLR1, TLR3, TLR7, or TLR9. Only 13 patients, however, fit this criterion. When we divided our 153 patients into two groups according to their TLR negativity for those four receptors, among those with negative immunoexpression, median survival time was 0.90 (95% CI 0.54–1.27; n = 13) years; among the rest, median survival time was 2.26 (95% CI 1.89–2.62; n = 140) years; p<0.001 (Fig 2).

In multivariate analysis, this phenomenon of poor prognosis persisted: those with TLR negativity had worse overall disease-specific survival (HR = 2.41; 95% CI 1.31–4.43; p = 0.005; n = 13) (Table 5). This held true especially for those patients with a lymph-node ratio of less than 20% (HR = 5.15; 95% CI 2.47–10.76; p<0.001; n = 10).

**Table 5:** Cox regression survival analysis of patients with pancreatic ductal adenocarcinoma related to clinicopathological characteristics and TLR1, TLR3, TLR5, TLR7, and TLR9 immunoexpression.

	N	Univariate analysis			Multivariate analysis		
		HR	95% CI	P value	HR	95% CI	P value
**TLR1**							
Negative (0)*	3	1.00			1.00		
Positive (1–3)*	151	0.50	0.16–1.58	0.235	0.38	0.12–1.22	0.105
**TLR3**							
Negative (0)	2	1.00			1.00		
Positive (1–3)	151	0.20	0.05–0.85	**0.029**	0.24	0.06–1.04	0.057
**TLR5**							
Negative (0)	30	1.00			1.00		
Positive (1–3)	123	0.80	0.52–1.23	0.315	0.72	0.47–1.12	0.143
**TLR7**							
Negative (0)	5	1.00			1.00		
Positive (1–3)	148	0.19	0.08–0.49	**0.001**	0.20	0.08–0.53	**0.001**
**TLR9**							
Negative (0)	7	1.00			1.00		
Positive (1–3)	146	0.42	0.20–0.91	**0.027**	0.57	0.26–1.28	0.175
**TLRs 1, 3, 7, and 9 combined**							
All positive (1–3)	140	1.00			1.00		0.444
Negative (0)	13	2.75	1.53–4.94	**0.001**	2.41	1.31–4.43	**0.005**

Univariate and multivariate Cox analysis. Multivariate analysis adjusted for age, gender, stage, lymph-node ratio, and post-operative adjuvant therapy.

*Numbers in parentheses indicate scoring intensity: (0) negative, (1) low, (2) moderate, or (3) strong.

In regard to TLR5, our analysis showed no prognostic value.

## Discussion

We found that strong TLR1 expression predicted better survival in PDAC, and that lack of expression of TLRs 1, 3, 7, or 9 indicated very poor survival. TLR expression pattern also differed from physiological expression. To our knowledge, no similar findings have yet appeared.

Prognostic roles of TLRs are contradictory. TLRs 7 and 9 have been speculated to accelerate tumor progression in pancreatic cancer [19–22], but clinical studies have shown TLR9 to be associated with better prognosis [23]. Our results support this latter hypothesis and reveal TLRs as indicators of better prognosis in PDAC. This is also in line with our earlier findings on TLR2 and TLR4 [18]. The prognostic roles of TLRs 1, 3, and 5 have not been studied previously in PDAC.

Plenty of evidence suggests a negative association between upregulated TLR expression and survival in other cancers. In gastric cancer, TLR2 and TLR9 are thought to account for the invasive abilities of *H*. *pylori*-mediated infection [24], and in papillary thyroid cancer, TLR3 overactivation is linked to cancer progression [25]. In cervical neoplasias, TLR5 is thought to promote tumor progression [26]. In lung cancer, TLR9 may be linked to metastatic properties [27], and TLR9 activation also stimulates prostate-cancer invasion [28]. This raises the question, why our results contradict those of many others, and whether different mechanisms are involved in different cancers.

Considering the physiological function of the TLRs, it is understandable why their expression is linked to worse survival. Tumor-induced necrosis produces cell debris which may activate the TLRs and result in a release of inflammatory cytokines, potentially creating a tumor-promoting microenvironment [6]. This would also explain the strengthened pancreatic carcinogenesis resulting from the presence of microbiota [12]. Although this seems logical, it does not help us understand how TLR expression would promote survival, as occurs in clinical PDAC studies.

Instead, a different mechanism may be at play. The function and effects of TLRs in PDAC may differ from their physiological function in infections [29]. The expression pattern in our samples was somewhat atypical. TLRs 3, 7, and 9 occur mainly in the cytoplasm during physiological activation, as was the case in our series. But interestingly, in our series, TLR1 was detected in the cytoplasm and TLR5 in both the nucleus and cytoplasm, whereas physiologically activated TLR1 and TLR5 are typically detected only on cell membranes. The meaning of this atypical expression among our findings raises speculation. All of the above implies that, under certain circumstances, non-physiological TLR activation may aid against tumor progression, and lack of TLR expression may indicate that this defensive role is not functioning.

One aspect of our patient selection also surfaced. In multivariate analysis, the connection between strong TLR1 expression and better prognosis was more evident in those patients who had received no post-operative adjuvant therapy. Naturally, there may be factors at play that were unmeasured in our analysis, factors defining which patients would receive adjuvant therapy and which would not. But if not, the result implies that the adjuvant therapy itself may affect the TLR1-modulated pathway, evening out the naturally occurring differences among patients. This would explain why the survival benefit was seen in those receiving no post-operative adjuvant treatment, and why such phenomenon was not apparent in those who did. But this also raises a question: to what extent do patients with strong TLR1 expression profit from the adjuvant therapy, and does a more suitable approach to treating this patient group exist?

Our study had some limitations. The most notable involves our methods, because immunohistochemistry evaluation is subjective by nature. To minimize any biased results, we had two independent observers examine the samples, and all differing scores were re-evaluated and discussed until consensus. The tumor microarray technique allows the use of a great number of patients. The downside is that only a limited area of the tumor is visible, allowing for sampling error. To prevent this, we took six cores from different parts of the tumor to maximize accuracy. Our patient cohort is inherently skewed, as it represented only those 15 to 20% of all PDAC patients who were resectable [3–5]. However, this also means that our patients shared a similar cancer stage and tumor microenvironment, which may prove crucial in identifying common prognostic markers, because the specific tissue microenvironment interaction may depend on tumor stage [30]. The patients negative for TLRs 1, 3, 7, or 9 were very few, but their poor prognosis stood out forcefully, and we trust that this finding reflects a true biological phenomenon.

Our biggest strength is our patient material. A population of 154 is relatively large, considering the low incidence of resectable PDAC. Furthermore, our patients were treated in 2002–2011, when both neoadjuvant and adjuvant therapy were less common. The few patients receiving neoadjuvant therapy we excluded altogether, as this treatment may affect the tumor microenvironment. Our patients gave us a unique chance to explore inflammation biomarkers in pristine tumor tissue.

In conclusion, we show that strong cytoplasmic TLR1 expression signifies positive prognosis in PDAC, and lack of expression of TLRs 1, 3, 7, or 9 indicates poor survival. More research will allow further elucidation of the role of toll-like receptors in various cancers.

## Supporting information

S1 Fig Staining patterns in lower magnification. (TIF)

S2 Fig Survival according to high and low number of scores per patient. (TIF)

S1 Table Number of cores per patient used for scoring. (DOCX)

S2 Table Number of cores used per patient for positive and negative scoring. (DOCX)

## References

[1] Bray F, Ferlay J, Soerjomataram I, Siegel RL, Torre LA, Jemal A. Global cancer statistics 2018: GLOBOCAN estimates of incidence and mortality worldwide for 36 cancers in 185 countries. *CA Cancer J Clin*. 2018;68(6):394–424. 10.3322/caac.21492 30207593

[2] Siegel RL, Miller KD, Jemal A. Cancer statistics. 2016. *CA J Clin*. 2016;66(1):7–30. 10.3322/caac.21332 26742998

[3] Hidalgo M. Pancreatic cancer. *N Engl J Med*. 2010;362(17):1605–17. 10.1056/NEJMra0901557 20427809

[4] Hidalgo M, Cascinu S, Kleeff J, Labianca R, Löhr JM, Neoptolemos J, et al Addressing the challenges of pancreatic cancer: Future directions for improving outcomes. *Pancreatology*. 2015;15(1):8–18. 10.1016/j.pan.2014.10.001 25547205

[5] Freelove R, Walling AD. Pancreatic cancer: Diagnosis and management. *Am Fam Physician*. 2006;73(3):485–92. 16477897

[6] Shacter E, Weitzman SA. Chronic inflammation and cancer. *Oncology (Williston Park)*. 2002;1–16. 11866137

[7] Quail DF, Joyce JA. Microenvironmental regulation of tumor progression and metastasis. *Nat Med*. 2013;19(11):1423–37. 10.1038/nm.3394 24202395 PMC3954707

[8] O’Neill LAJ, Golenbock D, Bowie AG. The history of Toll-like receptors—redefining innate immunity. *Nat Rev Immunol*. 2013;13(6):453–60. 10.1038/nri3446 23681101

[9] Li X, Jiang S, Tapping RI. Toll-like receptor signaling in cell proliferation and survival. *Cytokine*. 2010;49(1):1–9. 10.1016/j.cyto.2009.08.010 19775907 PMC2808458

[10] Sato Y, Goto Y, Narita N, Hoon DSB. Cancer cells expressing Toll-like receptors and the tumor microenvironment. *Cancer microenviron*. 2009;2(S1):205–14. 19685283 10.1007/s12307-009-0022-y PMC2756339

[11] Karin M. Nuclear factor-κB in cancer development and progression. *Nature*. 2006;441:431–436 10.1038/nature04870 16724054

[12] Thomas RM, Gharaibeh RZ, Gauthier J, Beveridge M, Pope JL, Guijarro MV, et al Intestinal microbiota enhances pancreatic carcinogenesis in preclinical models. *Carcinogenesis*. 2018;39(8):1068–1078. 10.1093/carcin/bgy073 29846515 PMC6067127

[13] Kelly MG, Alvero AB, Chen R, Silasi D-A, Abrahams VM, Chan S, et al TLR-4 signaling promotes tumor growth and paclitaxel chemoresistance in ovarian cancer. *Cancer Res*. 2006;66(7):3859–3868. 10.1158/0008-5472.CAN-05-3948 16585214

[14] Li X, Wang S, Zhu R, Li H, Han Q, Zhao RC. Lung tumor exosomes induce a pro- inflammatory phenotype in mesenchymal stem cells via NFκB-TLR signaling pathway. *J Hematol Oncol*. 2016;9:42 10.1186/s13045-016-0269-y 27090786 PMC4836087

[15] Wang EL, Qian ZR, Nakasono M, Tanahashi T, Yoshimoto K, Bando Y, et al High expression of Toll-like receptor 4/myeloid differentiation factor 88 signals correlates with poor prognosis in colorectal cancer. *Brit J Cancer*. 2010;102(5):908–915. 10.1038/sj.bjc.6605558 20145615 PMC2833250

[16] Mäkinen LK, Atula T, Häyry V, Jouhi L, Datta N, Lehtonen S, et al Predictive role of Toll-like receptors 2, 4, and 9 in oral tongue squamous cell carcinoma. *Oral Oncol*. 2015;51(1):96–102. 10.1016/j.oraloncology.2014.08.017 25264223

[17] Sato Y, Motoyama S, Wakita A, Kawakita Y, Liu J, Nagaki Y, et al TLR3 expression status predicts prognosis in patients with advanced thoracic esophageal squamous cell carcinoma after esophagectomy. *Am J Surg*. 2018;216(2):319–325. 10.1016/j.amjsurg.2018.01.038 29395019

[18] Lanki M, Seppänen HE, Mustonen HK, Böckelman C, Juuti AT, Hagström JK, et al Toll-like receptor 2 and Toll-like receptor 4 predict favorable prognosis in local pancreatic cancer. *Tumor Biol*. 2018;40(9). 10.1177/1010428318801188 30246618

[19] Grimmig T, Matthes N, Hoeland K, Tripathi S, Chandraker A, Grimm M, et al TLR7 and TLR8 expression increases tumor cell proliferation and promotes chemoresistance in human pancreatic cancer. *Int J Oncol*. 2015;47(3):857–66. 10.3892/ijo.2015.3069 26134824 PMC4532221

[20] Grimmig T, Moench R, Kreckel J, Haack S, Rueckert F, Rehder R, et al Toll like receptor 2, 4, and 9 signaling promotes autoregulative tumor cell growth and vEGF/PDGF expression in human pancreatic cancer. *Int J Mol Sci*. 2016;17(12):2060. 10.3390/ijms17122060 PMC5187860 27941651

[21] Ochi A, Graffeo CS, Zambirinis CP, Rehman A, Hackman M, Fallon N, et al Toll-like receptor 7 regulates pancreatic carcinogenesis in mice and humans. *J Clin Invest*. 2012;122(11):4118–4129. 10.1172/JCI63606 23023703 PMC3484447

[22] Wu HQ, Wang B, Zhu SK, Tian Y, Zhang JH, Wu HS. Effects of CPG ODN on biological behavior of PANC-1 and expression of TLR9 in pancreatic cancer. *World J Gastroenterol*. 2011;17(8):996–1003. 10.3748/wjg.v17.i8.996 21448350 PMC3057161

[23] Leppänen J, Helminen O, Huhta H, Kauppila JH, Isohookana J, Haapasaari KM, et al High Toll-like receptor (TLR) 9 expression is associated with better prognosis in surgically treated pancreatic cancer patients. *Virchows Arch*. 2017;470(4):401–410. 10.1007/s00428-017-2087-1 28191612

[24] Chang YJ, Wu MS, Lin JT, Chen CC. Helicobacter pylori-Induced invasion and angiogenesis of gastric cells is mediated by cyclooxygenase-2 induction through TLR2/TLR9 and promoter regulation. *J Immunol*. 2005;175(12):8242–8252. 10.4049/jimmunol.175.12.8242 16339564

[25] McCall KD, Harii N, Lewis CJ, Malgor R, Kim WB, Saji M, et al High basal levels of functional toll-like receptor 3 (TLR3) and noncanonical Wnt5a are expressed in papillary thyroid cancer and are coordinately decreased by phenylmethimazole together with cell proliferation and migration. *Endocrinology*. 2007;148(9), 4226–4237. 10.1210/en.2007-0459 17525119

[26] Kim WY, Lee JW, Choi JJ, Choi CH, Kim TJ, Kim BG, et al Increased expression of Toll-like receptor 5 during progression of cervical neoplasia. *Int J Gynecol Cancer*. 2008;18(2):300–305. 10.1111/j.1525-1438.2007.01008.x 17587322

[27] Ren T, Wen Z K, Liu Z M, Liang Y J, Guo Z L, Xu L. Functional expression of TLR9 is associated to the metastatic potential of human lung cancer cell: functional active role of TLR9 on tumor metastasis. *Cancer Biol Ther*. 2007;6(11):1704–1709. 10.4161/cbt.6.11.4826 17986857

[28] Ilvesaro J, Merrell M, Swain T, Davidson J, Zayzafoon M, Harris K, et al Toll like receptor-9 agonists stimulate prostate cancer invasion in vitro. *Prostate*. 2007;67(7):774–781. 10.1002/pros.20562 17373717

[29] Zhu J, Mohan C. Toll-like receptor signaling pathways–therapeutic opportunities. *Mediators Inflamm*. 2010(8):781235–7. 20981241 10.1155/2010/781235 PMC2963142

[30] Neesse A, Algül H, Tuveson DA, Gress TM. Stromal biology and therapy in pancreatic cancer: A changing paradigm. *Gut*. 2015; 64(9):1476–84. 10.1136/gutjnl-2015-309304 25994217

