# Supplementary figures and images for: Toll-like receptor 1 predicts favorable prognosis in pancreatic cancer
Source: PLoS One. 2019 Jul 17;14(7):e0219245. doi: 10.1371/journal.pone.0219245 (PMC6636725; doi:10.1371/journal.pone.0219245)

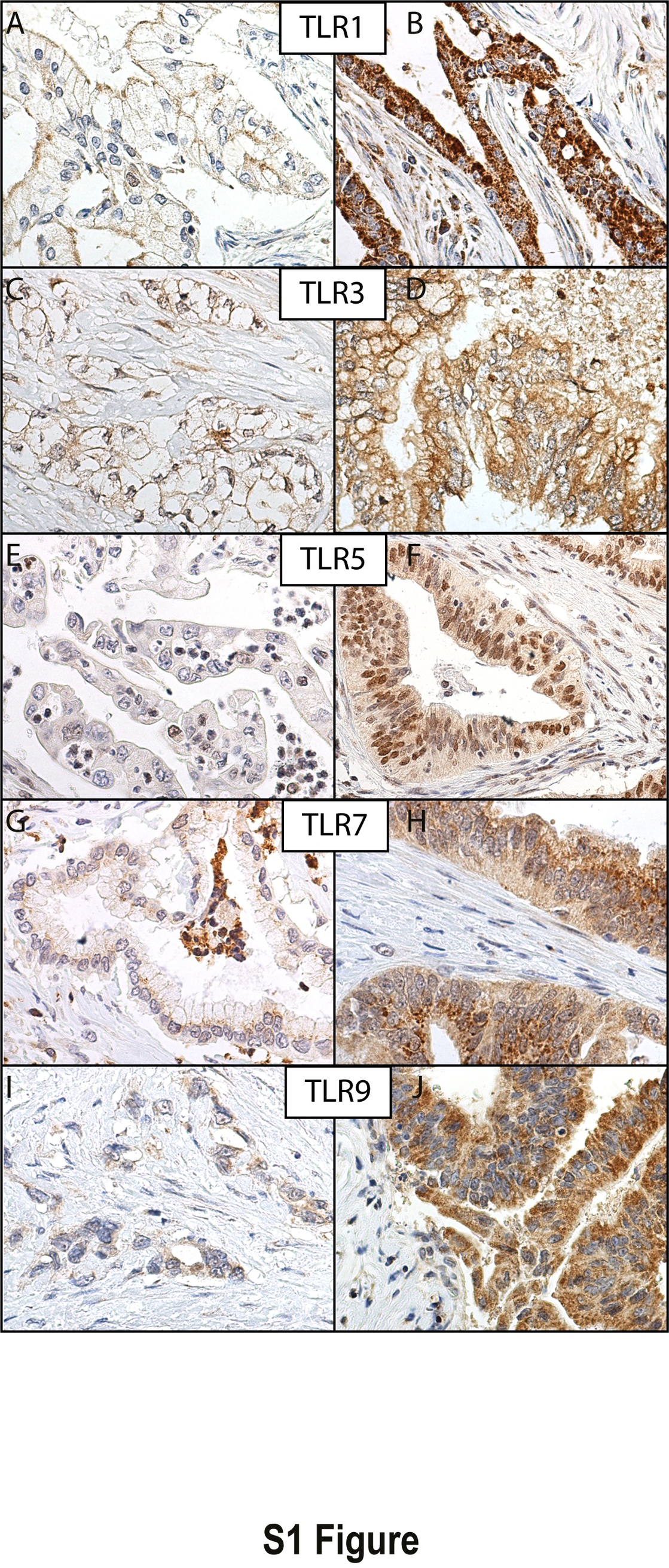

Supplement: S1 Fig — (TIF) [file pone.0219245.s001.tif]

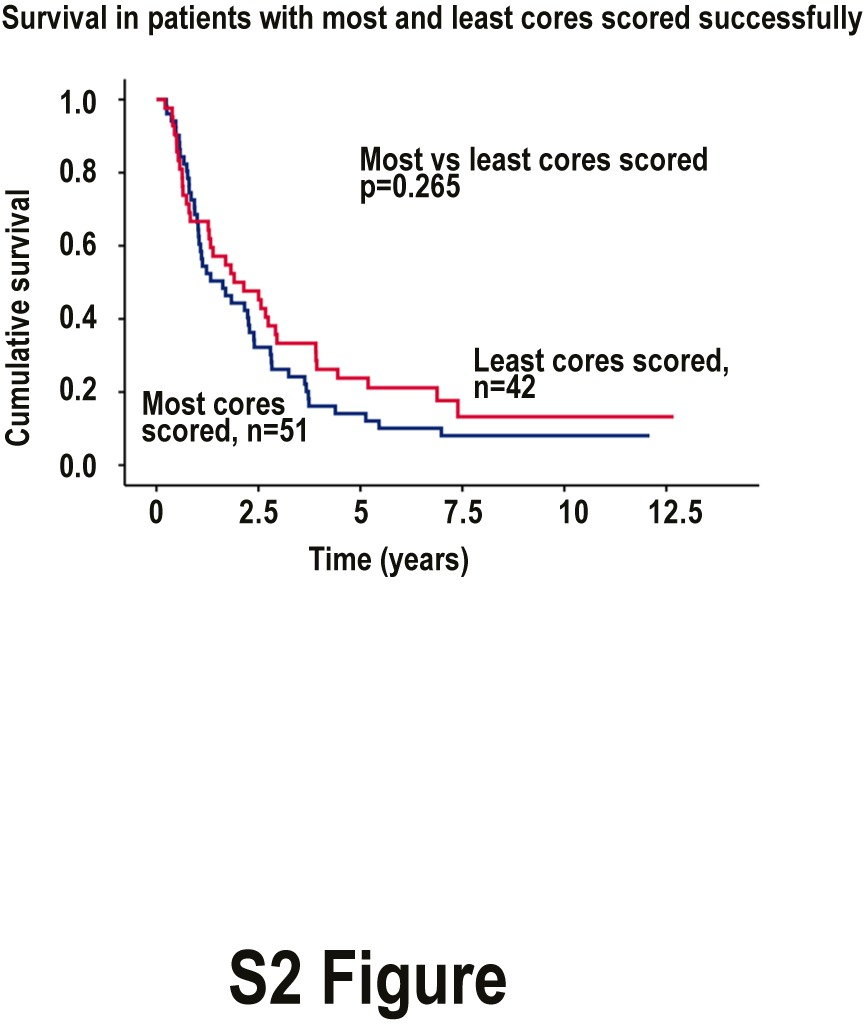

Supplement: S2 Fig — (TIF) [file pone.0219245.s002.tif]
