# Supplementary material for: Toll-like receptor 1 predicts favorable prognosis in pancreatic cancer
Source: PLoS One. 2019 Jul 17;14(7):e0219245. doi: 10.1371/journal.pone.0219245 (PMC6636725; doi:10.1371/journal.pone.0219245)
Supplement: S1 Table — (DOCX) [file pone.0219245.s003.docx]

**S1 Table. Number of cores per patient used for scoring**

| **Number of cores used** | **TLR1** | **TLR3** | **TLR5** | **TLR7** | **TLR9** |
| --- | --- | --- | --- | --- | --- |
| 6 or more | 81 | 84 | 91 | 80 | 76 |
| 5 | 34 | 34 | 28 | 33 | 35 |
| 4 | 25 | 22 | 20 | 27 | 28 |
| 3 | 8 | 8 | 8 | 5 | 9 |
| 2 | 3 | 2 | 5 | 4 | 4 |
| 1 | 3 | 3 | 1 | 4 | 1 |
