# Supplementary material for: Toll-like receptor 1 predicts favorable prognosis in pancreatic cancer
Source: PLoS One. 2019 Jul 17;14(7):e0219245. doi: 10.1371/journal.pone.0219245 (PMC6636725; doi:10.1371/journal.pone.0219245)
Supplement: S2 Table — (DOCX) [file pone.0219245.s004.docx]

**S2 Table. Number of cores used per patient for positive and negative scoring**

|  | **Number of patients** | **Cores per patient** | | | |
| --- | --- | --- | --- | --- | --- |
|  |  | Exact | Median | Lower quartile | Upper quartile |
| **TLR1** |  |  |  |  |  |
| 0 | 3 | 1, 4, 5 | - | - | - |
| 1-3 | 151 |  | 6 | 5 | 6 |
| **TLR3** |  |  |  |  |  |
| 0 | 2 | 1,5 | 5 | 4 | 6 |
| 1-3 | 151 |  | 6 | 5 | 6 |
| **TLR5** |  |  |  |  |  |
| 0 | 30 |  | 6 | 5 | 6 |
| 1-3 | 123 |  | 6 | 5 | 6 |
| **TLR7** |  |  |  |  |  |
| 0 | 5 | 6, 6, 5, 4, 2 |  |  |  |
| 1-3 | 148 |  | 6 | 4 | 6 |
| **TLR9** |  |  |  |  |  |
| 0 | 7 | 6, 5, 5, 4, 4, 3, 2 |  |  |  |
| 1-3 | 146 |  | 6 | 4 | 6 |
|  |  |  |  |  |  |
|  |  |  |  |  |  |
| **All TLR scores combined** | **154** |  | **27.5** | **23** | **30** |
